# Leveraging Digital Health to Improve the Cardiovascular Health of Women

**DOI:** 10.1007/s12170-023-00728-z

**Published:** 2023-09-30

**Authors:** Zahra Azizi, Demilade Adedinsewo, Fatima Rodriguez, Jennifer Lewey, Raina M. Merchant, LaPrincess C. Brewer

**Affiliations:** 1 Center for Digital Health, Stanford University, Stanford, CA USA; 2 Department of Cardiovascular Medicine and the Cardiovascular Institute, Stanford University, Stanford, CA USA; 3 Department of Cardiovascular Medicine, Mayo Clinic, Jacksonville, FL USA; 4 Department of Medicine, Division of Cardiology, University of Pennsylvania Perelman School of Medicine, Philadelphia, PA USA; 5 Center for Digital Health, University of Pennsylvania Perelman School of Medicine, Philadelphia, PA USA; 6 Department of Cardiovascular Medicine, Mayo Clinic College of Medicine, Rochester, MN USA; 7 Center for Health Equity and Community Engagement Research, Mayo Clinic, Rochester, MN USA

**Keywords:** Cardiovascular health, Clinical trials, Digital health, Equity, Prevention, Women

## Abstract

**Purpose of Review:**

In this review, we present a comprehensive discussion on the population-level implications of digital health interventions (DHIs) to improve cardiovascular health (CVH) through sex- and gender-specific prevention strategies among women.

**Recent Findings:**

Over the past 30 years, there have been significant advancements in the diagnosis and treatment of cardiovascular diseases, a leading cause of morbidity and mortality among men and women worldwide. However, women are often underdiagnosed, undertreated, and underrepresented in cardiovascular clinical trials, which all contribute to disparities within this population. One approach to address this is through DHIs, particularly among racial and ethnic minoritized groups. Implementation of telemedicine has shown promise in increasing adherence to healthcare visits, improving BP monitoring, weight control, physical activity, and the adoption of healthy behaviors. Furthermore, the use of mobile health applications facilitated by smart devices, wearables, and other eHealth (defined as electronically delivered health services) modalities has also promoted CVH among women in general, as well as during pregnancy and the postpartum period. Overall, utilizing a digital health approach for healthcare delivery, decentralized clinical trials, and incorporation into daily lifestyle activities has the potential to improve CVH among women by mitigating geographical, structural, and financial barriers to care.

**Summary:**

Leveraging digital technologies and strategies introduces novel methods to address sex- and gender-specific health and healthcare disparities and improve the quality of care provided to women. However, it is imperative to be mindful of the digital divide in specific populations, which may hinder accessibility to these novel technologies and inadvertently widen preexisting inequities.

## Introduction

In the digital revolution era, the world is transitioning to a more connected and technologically driven way of life. This transformation was accelerated by the COVID-19 pandemic requiring many organizations to adopt digital technologies and face new challenges associated with implementation and management of digital modalities [1]. Digital health (also known as electronic health or eHealth) is a broad term which refers to information technology devices and modalities used to manage patient health and promote wellness. The World Health Organization defines digital health as the field of knowledge and practice associated with the development and use of digital technologies to improve health [2]. These digital technologies include biometric monitoring technologies (e.g., fitness trackers, smartwatches, electrocardiogram (ECG) devices, oximeters, and portable blood pressure (BP) monitors) [3]. More recently, there has been a substantial increase in the use of personalized health tools including mobile health (mHealth) which utilizes mobile devices for exchange of information, communication, and data transfer between patients and clinicians [3]. These tools enable population-level interventions such as promoting health awareness and adoption of a healthier lifestyle, which is the cornerstone approach for primary prevention of cardiovascular diseases (CVDs) [4, 5].

CVD is the leading cause of morbidity and mortality among men and women worldwide. The 2019 Global Burden of Disease study estimated that 275 million women were living with CVD, with ischemic heart disease being the leading cause of cardiovascular death (47%) followed by stroke (36%) [6]. The age-adjusted CVD death rate in the USA has decreased to a larger extent for men than for women, and sex-based differences in cardiovascular physiology are known to exist [7]. However, CVD deaths have increased among younger women (< 65 years) in the USA [8]. As such, early prevention and targeted intervention strategies are essential to reducing CVD mortality and morbidity among women. In recent years, the information technology industry has made significant investments in health awareness targeted at encouraging consumers to engage in healthier lifestyles [9–13]. Devices, such as the Apple Watch® and Fitbit®, have created and leveraged incentive-based environments to provide feedback to end users and to reward healthy behaviors including exercise, sleep, and weight monitoring [13].

CVD among women is underrecognized, understudied, and undertreated [6], thus creating sex- and gender-based inequities in care which negatively influences patient outcomes [9, 10, 14, 15]. Historically, CVD was largely viewed as being a man’s disease which may be due to a higher incidence and prevalence of CVD among men compared to women [9, 10, 14–16]. Consequently, in most cardiovascular clinical trials, study participants are predominantly men and results are more reflective of this population [16–20]. Treatment recommendations for women are often based on these studies, although the scientific evidence may not necessarily be generalizable. A recent study by Alipour et al. [21] examined all published evidence presented in the most recent Canadian Atrial Fibrillation guidelines to determine if the studies have equal representation of men and women. This investigation demonstrated lower participation of women (39.1% overall) which was even lower in randomized controlled trials (RCTs) (33.8%). Additionally, this study found that many of the trials did not perform adequate sensitivity or sex- or gender-specific analyses to determine if the results were valid in women.

Women tend to have lower physical activity levels, a lower overall cardiovascular health (CVH) score, and higher prevalence of CVD risk factors at postmenopause leading to poorer CVD prognosis and outcomes compared to men [22]. A study investigating sex and gender differences and biases in artificial intelligence (AI) found that the underrepresentation of women in trials and healthcare data can result in suboptimal recommendations for women and hence hinder the potential benefits of AI integration in cardiovascular care [23].

In this review, we provide a comprehensive discussion on the population-level implications of digital health interventions (DHIs) and digital technologies on the CVH of women and strategies to optimally leverage these technologies for primary and secondary CVD prevention. The terms “males/men” and “females/women” are oftentimes incorrectly used interchangeably. However, in this review, we employ “male” and “female” to denote biological sex and “man” and “woman” to encompass gender, particularly in studies where these distinctions were not clearly delineated [22, 24, 25].

## Role of Digital Health to Promote Cardiovascular Health Through Prevention

Telemedicine has evolved from an experimental phase to large-scale adoption and implementation in the past few years. The COVID-19 pandemic led to rapid development and adoption of telemedicine and telehealth for CVD prevention [26]. The effective role of these modalities in CVD risk factor prevention has been examined in multiple clinical trials [27]. One RCT of 767 patients with heart failure (43.5% women) assessed the impact of short message service (SMS) for improving health outcomes and self-care behaviors (e.g., medication compliance, weight monitoring, diet, and exercise) [28]. The intervention reduced the composite endpoint of all-cause mortality and hospitalization (SMS: 50.4% vs. structured telephone support (STS): 41.3% vs. usual care group (UC): 36.5%, *P* < 0.05). There was also improved self-care behaviors within the SMS and STS groups compared to the control group (medication compliance, SMS: 78.9% vs. STS: 81.4% vs. UC: 69.5%, *P* = 0.011; water restriction, SMS: 70.8% vs. STS: 74.5% vs. UC: 61.5%, *P* = 0.013). A meta-analysis evaluating the effect of telehealth on major CVD outcomes demonstrated significant reduction in CVD incidence compared to usual care (RR, 0.59 [95% CI, 0.47–0.74]), although further investigations are needed to assess its effect specifically among women [29].

Numerous studies have demonstrated the effectiveness of digital health tools in reducing the burden of CVDs and improving outcomes by addressing multiple risk factors. A systematic review and meta-analysis by Widmer et al. [30] evaluated the impact of various DHIs including web-based consultations, mobile applications, and wearable devices. The authors concluded that DHIs significantly reduced the risk of adverse CVD outcomes (CVD events, all-cause mortality, hospitalizations) (risk ratio=0.61 (95% CI, 0.45–0.83), *P*=0.002), in addition to improving weight control (mean difference, −3.35 pounds (95% CI, −6.08 pounds, −1.01 pounds); *P*=0.006) [30]. In the MIPACT study by Golbus et al. [31], the authors aimed to monitor CVH behaviors obtained from wearable device signals in a large diverse population of men and women. All participants were provided with an Apple Watch® Series 3 or 4, an Omron Evolv® Wireless BP Monitor, and the MyDataHelps™ study smartphone application. Participants were instructed to wear their watch for at least 12 h per day and perform required daily and weekly tasks which included taking their BP and weight, respectively. The authors found adherence with these devices to be high (>98% completed study protocol) and that measures of physical activity among women were on average lower than men over 90 days follow-up in all age subgroups evaluated (number of steps: mean difference: men vs. women: 447 (95% CI 309–585; *P*<0.0001); walking distance (meters): mean difference: men vs. women: 731 (95% CI 603–859; *P*<0.0001)). As these devices become more ubiquitous in the population, the potential impact of this technology to facilitate CVD reduction interventions tailored for women could be substantial. For instance, setting and adhering to a daily goal for step count, calories burned, and sleep duration could reinforce healthy behaviors and digital health technologies can be leveraged to promote optimal CVH behaviors as defined by the American Heart Association Life’s Essential 8 framework [32].

Digital health technologies also provide a convenient method for remote monitoring by healthcare professionals which may directly impact CVH and outcomes. Senecal et al. tested a commercial mobile application-delivered weight loss program among 250,000 individuals (79% women) which consisted of dietary replacement, physical activity, and weight monitoring recommendations along with frequent guidance by program experts to assess weight loss maintenance. The authors found that although both men and women had significant weight loss, more women had a weight loss of at least 5% of their body weight compared to men (63.6% in women vs. 59% in men, *P*<0.001). Additionally, adherence and length of participation was significantly higher with women (121 days in women vs. 115 days in men; *P*<0.001) [33•]. Leveraging patients’ personal digital devices for remote coaching, and recommendation, in addition to providing feedback based on performance provides a potential avenue for healthcare professionals to motivate patients to adopt optimal CVH behaviors and prevent future CVDs [34].

The Health Information National Trends Survey from 2017 to 2020 evaluated digital health use among women who owned a smartphone or tablet (*N*=8573) with and without chronic disease (including CVD) [35]. Digital health use was captured by asking if women used their devices to track health goals, make health decisions, communicate with their healthcare provider or if they used a wearable device (e.g., Apple Watch® and Fitbit®). The authors found that those with multiple and chronic health conditions were more receptive to integrating digital health modalities in their daily lifestyle to manage their diseases compared to those with no chronic health conditions (OR 1.43, 95% CI 1.16–1.77; *P*=0.001). This study also highlighted specific subpopulations (including older, low income, and uninsured individuals) unable to take advantage of digital health tools mostly due to cost and limited availability of mobile and internet services [35]. Nevertheless, these findings underscore the importance and potential impact of digital integration for health promotion and improving disease outcomes. However, we must be aware that the digital divide can exacerbate health disparities and verify that measures are put in place to address digital equity prior to implementing DHIs on a broader scale [36].

The studies discussed above demonstrate the potential impact of digital technologies (including smartphones/tablets, wearable electronic devices, internet-based interventions, and telehealth) on CVH, particularly in women. Many of these technologies are now considered mainstream and have the potential to improve CVH at the population level and decrease the financial burden on the healthcare system [11, 13, 30, 35, 37–42, 43•, 44–46].

## Special Focus: Digital Health Interventions in Racial and Ethnic Minoritized Groups

The use of digital health applications has shown effectiveness in promoting CVH among racial and ethnic minoritized groups. A community-based, cluster, RCT among African American churches in Minnesota conducted by Brewer et al. tested an mHealth lifestyle intervention (FAITH! App) in improving CVH among predominantly African American women participants (71% of 85 total participants) [47•]. The intervention group demonstrated statistically significant improvements in overall CVH measured by the American Heart Association Life’s Simple 7 (LS7) score (1.9±1.9 in the intervention group vs. 0.7±1.7 in the control group, *P*<0.0001) at 6 months. Extrapolating from prior studies demonstrating that for each 1-point increase in LS7 score there is an estimated 19% and 11% reduction in CVD and all-cause mortality, respectively, the FAITH! App intervention [47•] could potentially yield benefits on overall longevity in study participants. In another study, Sutton et al. evaluated the role of mHealth in improving physical activity among African Americans (64% women) who participated in the Jackson Heart Study. The study found that using technology to track health was positively and significantly associated with favorable BP, body mass index, and cholesterol measurements in this population [48]. Additionally, a qualitative study using focus groups assessing components of an ideal mHealth weight management program for African American women concluded that they were receptive to these programs when culturally tailored to fit their specific needs [49]. As demonstrated by Joseph et al., a smartphone-based approach to improve physical activity among African American women may be feasible; however, attention to cultural and social support needs is essential to ensure maximal benefit in this population [50]. Similar themes were found when examining Hispanic populations in the USA. In their RCT, Linke et al. studied the benefits of physical activity programs delivered through mobile devices tailored to the participant culture and language [51]. The authors randomized 205 Latina women to either a culturally and linguistically tailored physical activity program or a general program on their website, to examine the use of their website and assess retention and participation in the program. The authors found that the tailored program resulted in more site visits (29 vs. 14.7, *P*<0.001) and more time spent on the website than the general program over 12 months of follow-up. Additionally, those in the tailored arm reached their physical activity goal more than the control group (achieving higher mean minutes of moderate to vigorous physical activity per week at 12-month follow-up: *b*=0.48, standard error (SE): 0.20, *P*=0.02) [51].

## Digital Health and Sex-Specific CVD Risk Factors

### Pregnancy-Related Studies

Technology and remote health interventions can be utilized to address CVD risk factors specific to women. For instance, a recent study by Sanghavi et al. investigated the role of telemedicine and rate of follow-up visit completion among postpartum patients with hypertension disorder of pregnancy or preeclampsia referred for postpartum hypertension management. The authors found that follow-up visit completion was significantly higher via telemedicine, particularly among Black women. The completion rate for traditional office visits was 32% compared to 70% for telemedicine (*P*<0.001) [52•], demonstrating the potential of telemedicine as a long-term modality to enhance healthcare delivery in vulnerable populations. It is well established that complications of pregnancy, including gestational diabetes, hypertension of pregnancy, preterm birth, and intrauterine growth restriction, increase the likelihood of future cardiometabolic diseases in the mother [53–55]. In their review, Jowell et al. investigated the efficacy of various DHIs and approaches in mitigating such risks in this population. Based on observational studies, the authors concluded that transitional clinics, in addition to lifestyle interventions, may reduce the incidence of type 2 diabetes mellitus and CVDs [56]. Both these methods can be implemented by eHealth approaches through the utilization of smart devices for follow-up.

Similar effects have been observed in postpartum women with hypertensive disorder of pregnancy. Lewey et al. randomized 127 participants to either an intervention (*N*=63, virtual teams with gamified approach including scores and rankings) or control (*N*=64, received daily feedback on goal attainment) group for 12 weeks. Participants in the intervention arm had a significantly higher increase in mean daily steps compared to the control (+Δ 647 steps; 95% CI, 169–1124 steps; *P*=0.009). In addition, higher proportions of participants in the intervention arm reached their daily steps goal [57•]. Another study examined the use of text-based remote monitoring in the management of 206 patients with postpartum hypertension [58]. Those randomized to the intervention (*N*=103) received home-based BP monitoring machines and text message reminders to follow their BP, whereas participants in the control arm (*N*=103) had standard-of-care follow-up. Those in the intervention group had a significantly higher rate of BP monitoring compared to the control group according to the American College of Obstetricians and Gynecologists criteria in 2013 [59] (defined as having a BP recorded on postpartum days 3–4 and 7–10) (92.2% vs. 43.7%; *P*<0.001). While there was no significant difference in the initiation of outpatient antihypertensive therapies or the number of office or emergency department (ED) visits for hypertension between the intervention and control groups, there was a statistically significant increase in hypertension-related readmissions in the control group compared to the intervention group (3.9 vs. 0%, *P*=0.04). Hence, this study demonstrates that the use of text messaging may allow for easier monitoring and higher adherence for this population compared to regular office visits. Further, Hirshberg et al. conducted a follow-up, retrospective cohort study of Independence Blue Cross members with a hypertensive disorder of pregnancy diagnosis enrolled in a postpartum remote BP monitoring program across three obstetric hospitals. Those enrolled in the program (*n*=1276) had fewer ED visits and hospital readmissions during the first 6 months after delivery when compared to patients that did not enroll (*n*=1276) (ED visits [*n* (%)]: text program: 21 (1.6%) vs. control: 36 (2.8%), *P*=0.04; inpatient readmissions [*n* (%)]: text program:17 (1.3%) vs. control: 38 (3%), *P*=0.005) [60]. None of these studies specifically assessed the role of the respective interventions on BP control.

The effect of an internet-based program on weight loss for postpartum women with low income was investigated in a cluster-randomized, single-blind, clinical trial of 371 patients [61]. Patients were randomized to either a 2-month primarily internet-based weight loss program (intervention group, *n*=174) or standard women, infants, and children (WIC) program (control group, *n*=197). Patients randomized to the intervention arm had greater mean 12-month weight loss (−3.2 kg in the intervention group vs. −0.9 kg in the standard-of-care group, *P*<0.001; difference, 2.3 kg (95% CI, 1.1 to 3.5)). Additionally, more participants in the intervention group were able to reach their pregestational weight (32.8% in the intervention group vs. 18.6% in the standard care group, *P*<0.001; difference, 14.2 percentage points (95% CI, 4.7 to 23.5)). This study demonstrates that internet-delivered programs may significantly improve postpartum weight loss in women of lower socioeconomic status. Hence, these investigations demonstrate the utility and benefits of leveraging digital health devices and services for improving CVH, particularly in women.

### Menopause-Related Studies

Internet-based coaching has shown promising results in hypertension control and abdominal obesity reduction in postmenopausal women [43•, 62]. In their study, Park et al. randomized 67 postmenopausal women to either the intervention group (*N*=34) which received daily SMS notifications over 12 weeks to encourage a healthier diet and regular physical activity or to the control group (*N*=33, no intervention delivered) [43•]. The study found that waist circumference (intervention: −3 cm, *P*=0.001, control +0.9, *P*=0.001), body weight (intervention: −2 kg, *P*=0.001, control: +0.7, *P*=0.001) and BP (intervention: −6.5 systolic and −4.6 diastolic, *P*=0.001, control: +0.9 systolic and +1.5 diastolic, *P*=0.001) measures significantly improved in the intervention group compared to the control.

### Digital Health Interventions to Increase Representation and Participation of Women in Cardiovascular Clinical Trials

Women particularly those from racial and ethnic minoritized groups are less likely to be enrolled in cardiovascular clinical trials [63]. There are a number of barriers which potentially account for this issue including differential care, ageism, logistical barriers, and systemic factors including discrimination [64, 65]. Increasing diversity and enrollment of women in clinical trials not only improves generalizability of clinical trials but also advances health equity and justice. Inclusion of diverse patient populations allows for development of treatment approaches which improves outcomes for all women [16, 17, 19, 20, 62, 63, 66, 67, A systematic review by Khan et al. [62], sought to evaluate the participation of older women in clinical trials investigating the effects of lipid-lowering therapies. Although enrollment of women has increased over time (since the 1993 NIH Revitalization Act and the establishment of the NIH Office of Research on Women’s Health), they remain underrepresented in the majority of these clinical trials. This limits the evidence base for efficacy and safety of lipid-lowering agents for women [62]. Based on the most recent Food and Drug Administration clinical trial data, nearly 70% of participants in cardiovascular clinical trials are men, whereas other fields including gastroenterology, oncology, and endocrinology have a fairly even distribution [20].

Disruption of clinical trials during the COVID-19 pandemic led to development of a novel approach for clinical trial administration [68]. Decentralized clinical trials also denoted as “direct-to-participant trials” are studies aimed to approach, enroll, and follow-up with patients directly without the need to visit a local enrolling site. Instead, a virtual singular coordinating center with the aid of a patient’s smartphone and internet connectivity is utilized [68]. All data is obtained through smart devices and transmitted to the coordinating center, while all follow-up visits are completed virtually [68]. This approach has many benefits including cost reduction and improved accessibility, in addition to improved external validity of the findings. Though there are challenges with decentralized trials, including users’ ability to interact with smart devices and digital literacy levels, availability of internet connectivity, and patient data privacy concerns, these trials can effectively mitigate geographical, structural, and financial barriers for study participant enrollment.

Though the adoption of digital methods in clinical trials has been slow, the COVID-19 pandemic led to interruptions and difficulties with enrollment in many clinical trials. Thus, the need for decentralized trials supported by digital health technologies became more urgent. Multiple recent studies have demonstrated the feasibility of digitally enabled, fully decentralized and pragmatic trials in the field of cardiovascular medicine [69–71]. Successful implementation of decentralized clinical trials requires collaborative efforts from all key stakeholders including patients, coordinating centers, and sponsoring organizations [47•, 72, 73]. While some aspects of clinical trials may require study participants to visit a site (e.g., for testing, imaging, or physical exams), many other requirements for participation could be conducted remotely. Thus, decentralized clinical trials could potentially create more opportunities for women to participate in studies by recognizing and circumventing responsibilities traditionally attributed to women (e.g., caregiver roles and household maintenance) [12]. For instance, a randomized trial for smoking cessation by Watson et al. [74] compared traditional (non-digital) versus web-based survey panel methods for recruitment (11.35% traditional vs. 88.65% digital). The trial successfully enrolled 79% women participants with high retention rate from diverse geographical and ethnic distribution, likely given the incorporation of primarily online, incentivized surveys. Thus, by leveraging digital technology, the authors were able to improve enrollment, representation, and retention of women.

Several studies have demonstrated the potential of digital technologies to enhance recruitment of historically marginalized women in clinical trials and research. This becomes particularly important among women from historically marginalized communities. The FAITH! Trial harnessed digital health to promote recruitment of African American women into the community-based study through community-informed digital recruitment (e.g., social media and electronic flyers with QR codes to study materials) [47•]. James et al. demonstrated that culturally tailoring of eHealth or mHealth research can encourage participation of African American women [75, 76]. They found that older women were motivated to participate in these studies if referred by a healthcare provider. They also noted that a key barrier to participating in mHealth research was access to a smartphone and mobile data which may hinder equitable access to these types of studies. In another study, Liao et al. sought to investigate the perception of an mHealth intervention for physical activity and sleep among African American women [77]. Based on respondent surveys, the authors concluded that mHealth delivery method may be a viable behavioral change strategy to promote increased physical activity and improvement of sleep among African American women, given their willingness to participate and utilize such technologies.

## Challenges and Barriers

In the sections above, we discussed the potential benefits of digital health integration for preventing CVD and improving CVD outcomes. However, there are potential challenges and barriers to adoption of DHIs. A substantial financial cost [78] can create barriers to digitally delivered healthcare for patients with lower socioeconomic status as well as racial and ethnic minoritized groups [73, 79, 80]. The digital divide refers to disparities created by unequal access to technology and internet connectivity due to financial, physical, or intellectual limitations [78]. According to a Pew Research Center survey of US adults in 2021, a disparity persists between Americans with lower and higher household incomes in relation to technology adoption and online access [81]. Eberly et al. [78] evaluated 148,402 patients scheduled for telemedicine visits during the pandemic and found that older age, female sex, Black race, Hispanic ethnicity, and lower household income were associated with less video use for telemedicine. Mariscal et al. highlighted that sociocultural norms may impact digital inclusion efforts among women and based on global data show that mobile phone and internet use was lower among women compared to men particularly in low- and middle-income countries [82]. Digital inclusion has been referred to as a key social determinant of health [36, 83] and should be investigated in conjunction with other known digital determinants of health to better understand its impact on cardiovascular outcomes and health inequity [10, 36, 84–86•].

Other barriers to digital health use include concerns regarding patient data privacy, lack of trust, and unwillingness to share personal digital information [87]. A national survey demonstrated that US adults were reluctant to share their digital data for health-related uses, highlighting the importance of having privacy protections in place to increase consumer trust [87]. Finally, varying digital literacy at individual and community levels introduces challenges in integrating DHIs in diverse populations. This is often seen in older populations and persons with low socioeconomic status who do not regularly interact with smartphones or other device-based technologies given limited availability of resources [44, 76].

## Future Directions

**Fig. 1:**
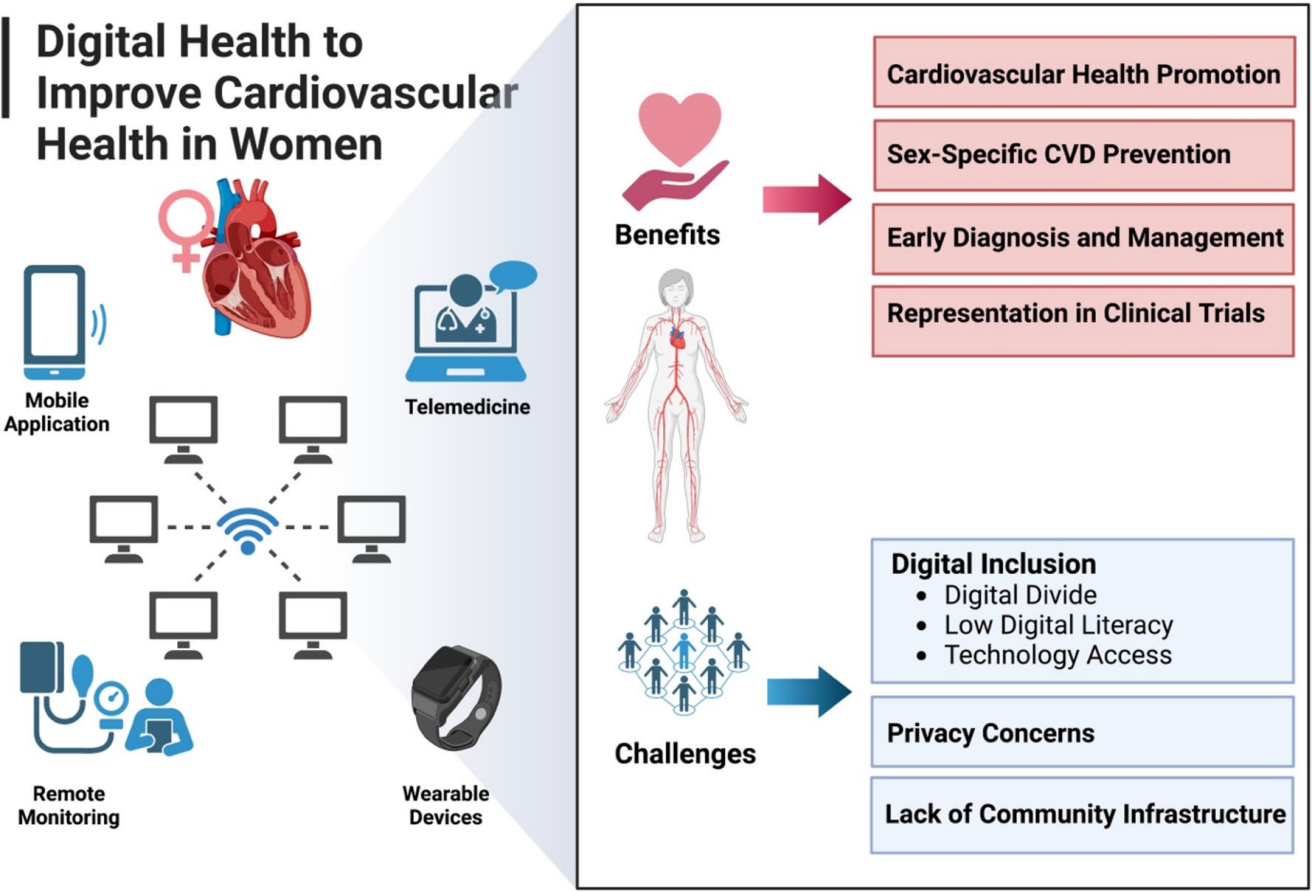
Leveraging digital health to improve cardiovascular health in women. Leveraging digital health and its various tools including artificial intelligence can promote cardiovascular health, improve cardiovascular health through prevention-focused interventions, and provide more equitable access and representation among women in clinical trials. This requires building national and community-level infrastructures to address the digital divide and digital health inequities

The use of digital devices and AI methods can potentially ensure more equitable access to care and clinical trial participation among women (Fig. 1) [9, 73]. There is increasing recognition of the importance of including women by technology industries which has led to the development of technology solutions specifically for women termed “femtech” [88, 89]. It is essential that all key stakeholders, and not women alone, are involved in order to fully take advantage of digital technologies to improve CVH in women. These stakeholders include but are not limited to technology companies who play a major role in the development of digital devices, researchers who design and conduct cardiovascular clinical trials, funding agencies who determine what type of research is funded, state and federal government who can ensure equitable access to digital technologies through appropriate laws and policies, and health insurance companies who can ensure adequate coverage of care delivered via digital technologies by health plans.

This collaborative effort is especially important as the move toward healthcare digitization accelerates and becomes adopted more broadly. There are multiple opportunities for exploration of the utility of DHIs among women. Adedinsewo et al. discuss opportunities to utilize digital tools and AI for cardiovascular screening throughout a woman’s life course [9]. However, fewer studies have evaluated the use of AI or machine learning models specifically among women, particularly Black women who are disproportionately affected by CVD. Many studies using machine learning methods in cardiovascular medicine lack external validation, have yet to be evaluated prospectively, or are in clinical trials [90]. These research gaps need to be addressed before we can truly realize the potential of digital health for improving women’s health. Rigorous and targeted interventions at the individual, institutional, state, and federal levels in a diverse group of women are essential for adoption and integration of digital health tools with the overarching goal of advancing health equity among women. Therefore, future research is warranted to understand disparities in outcomes by race, ethnicity, and urban/rural settings.

## Conclusion

In conclusion, the incorporation of digital technologies and strategies presents a promising avenue for tackling sex- and gender-specific health and healthcare disparities, ultimately enhancing the quality of care offered to women. Nevertheless, we must acknowledge and address the digital divide that exists in certain populations, as it may impede access to these innovative technologies and unintentionally exacerbate existing inequities. By taking proactive measures to bridge this gap and ensure inclusivity, we can truly harness the full potential of digital advancements to promote better CVH outcomes for all women.

## Abbreviations

AI: Artificial intelligence
BP: Blood pressure
CVD: Cardiovascular disease
CVH: Cardiovascular health
DHI: Digital health intervention
eHealth: Electronic health
ECG: Electrocardiogram
ED: Emergency department
LS7: Life’s Simple 7
mHealth: Mobile health
RCT: Randomized clinical (or controlled) trial
SMS: Short message service
